# Educating Women About Their Rights After Gender-Focused Legislation can Empower Mothers and Working Women

**DOI:** 10.3389/ijph.2022.1605231

**Published:** 2023-01-06

**Authors:** Mónica María Cortés Gallego

**Affiliations:** ^1^ Faculty of Health Sciences- Nursing Program, University of Quindío, Armenia, Colombia; ^2^ Nursing Sciences (Doctoral School), University of Jaume I, Castelló de La Plana, Spain

**Keywords:** women, women empowerment, human rights, legislation, education impacts, mother

**The IJPH series “Young Researcher Editorial” is a training project of the Swiss School of Public Health.**

Legislation is used to protect the population, increase equity, and reduce vulnerability to the adversities of the environment. Women have gained their rights through years of struggle and have forced many countries to recognize that laws are necessary to guarantee their equal participation in the social, economic and political aspects of society. But many people are unaware of women’s rights [1], which hinders women’s access to benefits they have the right to claim. According to United Nations Women, (ONU *mujeres*, UN Women), women must be informed about their rights and protections so they can claim them. This knowledge may begin to change social stereotypes that reduce gender equality and women’s conscientious, free, and informed decision-making [2].

Gender-focused legislation is written to overcome the economic, political, and cultural differences that separate men and women, exclude women, and foster social inequality [3] The feminist practice of “consciousness raising” captures a vision of women’s empowerment in which education expands consciousness and encourages women to act to transform their worlds [4].

But being a woman still hinders women in entering the labor market. The report on the social panorama of Latin America 2021 reported that women between 25 and 59 years old have a higher rate of poverty than men in the same age bracket [5]. The International Labor Organization (ILO) 2021b report, which places the region of the Americas far fewer women are employed and the informal employment rate is over 80% [6]. Thus, although women’s rights are better understood and defended today, they must become a reality for all women [2]. In places with high poverty rates, as it has been reported for countries like Colombia in regions such as Choco, Guajira or Orinoquia women can hardly finish secondary and university studies. Despite being a constitutionally recognized fundamental right.

Motherhood is also a source of inequality: protections granted by law are often not honored in practice [7]. Where, finally, motherhood often derails women’s careers. Being a mother at a young age can, interrupt their schooling and the stigma around being a young mother makes them vulnerable. Often, they are socially excluded and living in conditions of extreme poverty [8].

Initiatives like extending maternity leave, programs that prevent adolescent pregnancy, specific legislation for human conditions for giving birth, allocating time for breastfeeding after returning to work, providing lactation rooms [7], and other policies designed to support parental co-responsibility increase equal opportunities for women. But these initiatives must be accompanied by outreach and education about women’s labor, reproductive, and health rights. Women need this information to make decisions about motherhood, abortion, and family planning. A woman who understands her own physiology and knows her reproductive rights is more likely to be able to delay pregnancy if she wishes, and to take advantage of greater social and economic opportunities [9]. A mother who knows her rights is more likely to assert them and remain active in other social roles.

Economic empowerment of women is more than a human rights issue, and more than a struggle to participate on equal terms with men in the labor market. Economic empowerment brings self-sufficiency and economic efficiency; societies benefit from utilizing the skills of women who seek personal, family, and social benefits through work. Education and a formal job can give women access to a steady salary, and the kind of dignity that raises their quality of life. Educating more women will grow the economy and reduce infant mortality about 9.5% for each additional year of training [3].

Motherhood may be a key and sought-after role for many women, but it is not everything to all women. Women’s are in constantly debating not to disappear as a subjects [10]. Societies must inform women about the rights and initiatives that were designed to increase and ensure these initiatives are enacted in real life, so that they improve women’s quality of life and their health, and open opportunities for women to exercise sovereignty over their bodies while expanding their role in society.
